# Oral probiotic and prevention of *Pseudomonas aeruginosa *infections: a randomized, double-blind, placebo-controlled pilot study in intensive care unit patients

**DOI:** 10.1186/cc6907

**Published:** 2008-05-20

**Authors:** Christiane Forestier, Dominique Guelon, Valérie Cluytens, Thierry Gillart, Jacques Sirot, Christophe De Champs

**Affiliations:** 1 Université de Clermont 1 UFR Pharmacie Laboratoire de Bactériologie, 28 place Henri Dunant 63000 Clermont-Ferrand France; 2 CHU Clermont-Ferrand, Hôpital Gabriel Montpied, Service de Réanimation médico-chirurgicale 63000 Clermont-Ferrand, France; 3 Université de Clermont 1 UFR Médecine CHU Clermont-Ferrand, Hôpital Gabriel Montpied Laboratoire de Bactériologie, 63000 Clermont-Ferrand France; 4 Laboratoire de Bactériologie-Virologie-Hygiène CHU Robert Debré de Reims and UFR Médecine Université Reims Champagne-Ardenne, 51092 REIMS France

## Abstract

**Introduction:**

Preventing carriage of potentially pathogenic micro-organisms from the aerodigestive tract is an infection control strategy used to reduce the occurrence of ventilator-associated pneumonia in intensive care units. However, antibiotic use in selective decontamination protocols is controversial. The purpose of this study was to investigate the effect of oral administration of a probiotic, namely *Lactobacillus*, on gastric and respiratory tract colonization/infection with *Pseudomonas aeruginosa *strains. Our hypothesis was that an indigenous flora should exhibit a protective effect against secondary colonization.

**Methods:**

We conducted a prospective, randomized, double-blind, placebo-controlled pilot study between March 2003 and October 2004 in a 17-bed intensive care unit of a teaching hospital in Clermont-Ferrand, France. Consecutive patients with a unit stay of longer than 48 hours were included, 106 in the placebo group and 102 in the probiotic group. Through a nasogastric feeding tube, patients received either 10^9 ^colony-forming units unity forming colony of *Lactobacillus casei rhamnosus *or placebo twice daily, from the third day after admission to discharge. Digestive tract carriage of *P. aeruginosa *was monitored by cultures of gastric aspirates at admission, once a week thereafter and on discharge. In addition, bacteriological analyses of respiratory tract specimens were conducted to determine patient infectious status.

**Results:**

The occurrence of *P. aeruginosa *respiratory colonization and/or infection was significantly delayed in the probiotic group, with a difference in median delay to acquisition of 11 days versus 50 days (*P *= 0.01), and a nonacquisition expectancy mean of 69 days versus 77 days (*P *= 0.01). The occurrence of ventilator-associated pneumonia due to *P. aeruginosa *in the patients receiving the probiotic was less frequent, although not significantly reduced, in patients in the probiotic group (2.9%) compared with those in the placebo group (7.5%). After multivariate Cox proportional hazards modelling, the absence of probiotic treatment increased the risk for *P. aeruginosa *colonization in respiratory tract (adjusted hazard ratio = 3.2, 95% confidence interval – 1.1 to 9.1).

**Conclusion:**

In this pilot study, oral administration of a probiotic delayed respiratory tract colonization/infection by *P. aeruginosa*.

**Trial registration:**

The trial registration number for this study is NCT00604110.

## Introduction

Hospital-acquired infections are recognized as an important determinant of outcome in patients who require intensive care unit (ICU) admission. The source of respiratory tract colonization can be exogenous, for example from the hands of health care workers or the patient's skin, but it can also be endogenous, such as from the intestine, the oropharynx and the gastric compartment, followed by retrograde contamination [1-4]. Bacterial proliferation in the stomach is potentially enhanced by enteral nutrition in combination with administration of anti-ulcer prophylaxis drugs, which jeopardize the physiological barrier of the gastric compartment by buffering the gastric content and thereby facilitate bacterial proliferation.

Although the relative impact of gastric colonization on the occurrence of both early and late-onset ventilator-assisted pneumonia (VAP) is controversial [4-9], selective decontamination of the digestive tract (SDD) has been used by some authors as an infection prophylaxis strategy [10-12]. SDD is a four-component strategy and typically includes enteral nonabsorbed antimicrobial drugs applied to throat and gut throughout the ICU stay to control aerobic Gram-negative bacilli, yeasts and *Staphylococcus aureus*; a parenteral antibiotic given immediately on admission to prevent primary endogenous early infections; together with a high standard of hygiene to control transmission of pathogens and surveillance samples of throat and rectum to monitor the efficacy of treatment [13]. This approach aims to eradicate colonization of potentially pathogenic aerobic micro-organisms from the oropharynx, stomach and gut, while leaving the indigenous anaerobic flora largely undisturbed. Several recent meta-analyses [14-16] have shown that SDD significantly reduces infections in ICU patients, but the selective pressure exerted by antibiotic can lead to a dramatic adverse effect, namely overgrowth of members of the indigenous microflora or of ingested pathogens resistant to the agents administered [17-20]. Some studies have highlighted the controversy in this area [21,22], new attempts to inhibit intestinal or gastric colonization by pathogens should be assessed, and the World Health Organization has advocated the use of microbial interfering nonpathogens (probiotics) to restrain pathogens by impairing the colonization of mucosal surfaces [23].

Probiotics are defined as nonpathogenic bacteria that are allochthonous to the bacterial community of the digestive tract. Most bacterial probiotics are strains of the lactic acid bacteria *Lactobacillus*. By creating an indigenous microflora with bacteria that are well adapted to acid environments and known to prevent the growth of non-acid-tolerant bacteria, the barrier function could be reinforced and would help to prevent nosocomial infections associated with gut contamination. To test this hypothesis, we conducted a prospective double-blind randomized study in ICU patients to assess the impact of enteral administration of *Lactobacillus casei rhamnosus *strain 35 (Lcr35), a well documented probiotic strain that is manufactured as a pharmaceutical product [24,25], on gastric and respiratory tract colonization/infection by *Pseudomonas aeruginosa*. The latter micro-organism is the most commonly isolated antibiotic-resistant Gram-negative bacteria in VAP, and it is associated with significant morbidity and mortality rates [26-28].

## Materials and methods

### Patients and setting

Patients aged 18 years or older with a stay longer than 48 hours and a nasogastric feeding tube were eligible for inclusion in the study. Patients with any of the following were excluded: age under 18 years, immunosuppression, absolute neutrophile count under 500/mm^3^, gastrointestinal bleeding, contraindication to enteral feeding, and isolation of *P. aeruginosa *from gastric aspirates or respiratory tract specimens during the first 4 days after admission. The study was conducted in one 17-bed ICU in the teaching hospital of Clermont-Ferrand, France between March 2003 and October 2004. The study complied with the Helsinki Declaration and received ethical approval from the Comité Consultatif de Protection des Personnes dans la Recherche Biomédicale d'Auvergne (CCPRB, AU 479). Before inclusion in the study, patients or their closest relative provided written informed consent.

### Probiotic administration

Patients were administered *L. casei rhamnosus *(10^9 ^colony-forming units; the available pharmaceutical form no E01-A02-S06) or placebo (growth medium without bacteria) twice daily through a double-lumen nasogastric suction tube (Maxter-catheters, Marseille, France) or orally, after removal of the tube, from the third day after admission to the ICU until discharge or death.

### Objectives

The primary objective of the study was to determine whether probiotic administration delayed *P. aeruginosa *colonization in the gastric and respiratory tracts. An increase in the number of *P. aeruginosa *organisms was observed in the ICU during the year when the trial was designed. Most studies report that *P. aeruginosa*, and especially multiresistant *P. aeruginosa*, are generally isolated after a long stay in hospital that includes a period of ventilatory assistance of longer than 7 days [3,20,27,29-31]. Hence, delaying *P. aeruginosa *colonization would prevent *P. aeruginosa *infection.

The secondary objectives were to determine whether probiotic administration delayed respiratory tract infection or colonization due to *P. aeruginosa*, and to evaluate the ability of *L. casei rhamnosus *to persist in the stomach.

### Outcomes

The primary outcome was the time of first *P. aeruginosa *acquisition. The secondary outcomes were the times of *P. aeruginosa *respiratory tract infection or colonization and *P. aeruginosa *gastric colonization, and the number of patients with persistent gastric colonization with *L. casei rhamnosus*.

### Sample size

The number of patients required to achieve sufficient power for statistical analysis in this study was determined, assuming that the mean time to *P. aeruginosa *acquisition would be 15 days and considering that a 7-day increase in this time would be beneficial in terms of prevention, given the median length of stay. On the basis of this hypothesis, in order to compare the two groups with the log-rank test, for a significance level α = 0.05 and a power 1 – β = 0.90, 11 patients with *P. aeruginosa *would have been required in each group. Our ICU records from previous years indicated that about 8/100 patients acquired a *P. aeruginosa *strain. Hence 150 patients were required in each group, and so we set a target of 200 patients for each group.

### Randomization

Equal randomization to one of the two treatment arms was done using a computer-generated random allocation schedule. Envelopes numbered 1 to 400 contained the letter 'A' or 'B'.

Placebo and probiotics were manufactured by Lyocentre (Aurillac, France) and labelled 'A' or 'B'. Equal randomization to one of the two treatment was done by a computer-generated random allocation of envelopes numbered 1 to 400 and containing the letter 'A' or 'B'.

On the third day of hospitalization, when a patient met the inclusion criteria, the nurse opened the envelope following the numerical order and started the indicated treatment. The list of patients, their number of enrollment and their group were given to the bacteriology laboratory for statistical analysis at the end of the study.

### Statistical analysis

Evaluation criteria were the rates of gastric *P. aeruginosa *colonization and respiratory tract infection or colonization. For statistical analysis, we used the SPSS 11.0 program (SPSS, Paris, France). χ^2 ^or two-tailed Fisher exact test were used to compare qualitative variables and Student's *t*-test or Mann-Whitney test for quantitative variables. The geometric means of *Lactobacillus *concentrations in gastric aspirates were calculated for each patient. The results were then expressed as the medians of the means obtained for the all patients who were positive for Lcr35 in gastric aspirates. Statistical significance was established at *P *< 0.05. The mean nonacquisition expectancy (length of stay without *P. aeruginosa *acquisition) was calculated, and *P. aeruginosa *noncolonized patient rates were estimated and the two groups compared with regard to survival curves from grouped data using the Kaplan Meier method and the log-rank test. We looked for independent risk factors of *P. aeruginosa *acquisition by means of a step-wise Cox proportional hazards model. This model assessed the effect of each predictor on the hazard rate of occurrence over time, after adjustment for other factors and after allowing for censoring because of discharge, death and loss to follow up. We used graphical methods to check the proportional hazards assumption. Continuous variables that did not satisfied the linearity assumption were dichotomized. Variables for which *P *was 0.1 or less in the simple Cox regression analysis were entered into the multivariable analysis. The strength of the association between prognostic variables, and the outcome of interest was expressed as a hazard ratio and corresponding 95% confidence interval (CI) calculated [32,33].

### Definition and microbiological techniques

The following clinical data were recorded: age, sex, the Simplified Acute Physiologic Score (SAPS II) [34], underlying diseases and previous antibiotic treatments. Throughout the course of the study, administration of antibiotics and bacteriological data were recorded. VAP was defined according mostly to the US Centers for Disease Control and Prevention's National Healthcare Safety Network criteria [35]. These criteria require there to be at least one positive sample (protected specimen brush or plugged telescoping catheter for bronchoalveolar minilavage [>10^3 ^colony-forming units (CFUs)/ml] or endotracheal aspirate with [>10^5 ^CFUs/ml and >25 leucocytes/high-power field]) [27]; also required is the presence of one or several new abnormal radiographical and progressive parenchymatous infiltrates and one of the following signs: purulent sputum production, fever (temperature > 38.5°C), pathogenic bacteria in blood culture without other infection source, and bronchoalveolar minilavage with more than 5% cells with intracellular bacteria. The bronchoalveolar minilavage was performed by instilling 20 ml sterile physiological saline solution through a mini-PBAL catheter (Combicath; Plastimed Lab; Saint Leu La Forêt, France) [36].

The presence of *P. aeruginosa *was detected in gastric aspirates collected at admission, once a week as long as the gastric tube was present, and at discharge. In patients receiving enteral nutrition, gastric aspirates were taken before feeding bootle change (12 hours after probiotic administration). When no gastric residue was obtained, 10 ml physiological saline was injected into the tube and aspirated. Gastric aspirates were plated onto Drigalski with and without incorporated ceftazidime (4 mg/l) and were tenfold serial diluted before inoculation of MRS agar (Oxoïd, Basingstoke, England) for numbering *L. casei rhamnosus *CFUs. Bacterial isolates were identified with ID32GN API System (BioMérieux, Marcy l'Etoile, France). Findings of analyses of specimens taken for microbiological diagnosis because of patient infectious status (routinely performed by the hospital laboratory) are included in the study.

## Results

**Figure 1:**
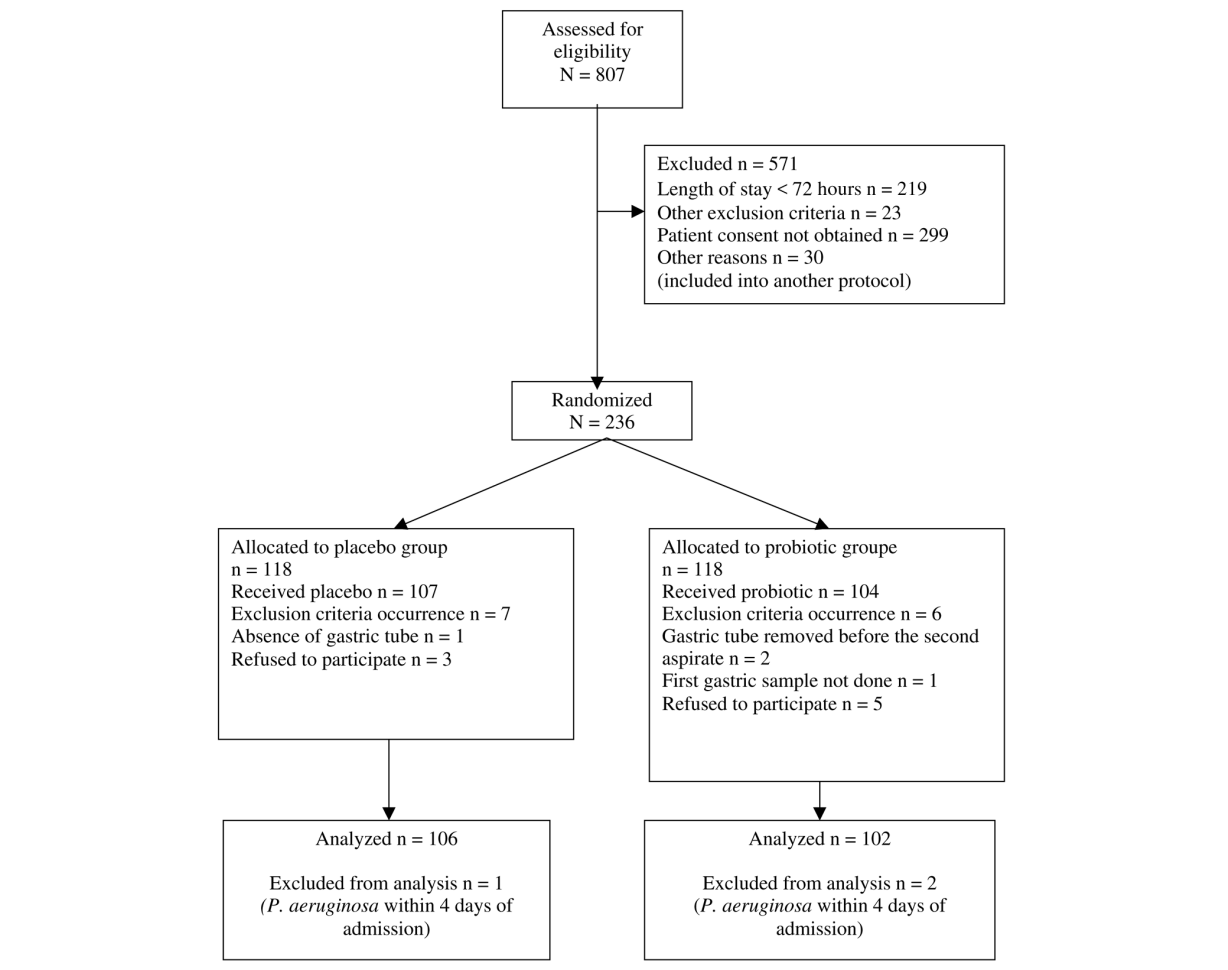
Patient flowchart.

A total of 807 patients were admitted in the unit during the period of the survey; 571 were not randomized: 242 because they did not meet inclusion criteria (219 stayed < 48 hours), 299 because patient consent was not obtained, and 30 because the patients were included in another protocol. After randomization, 28 were excluded because of occurrence of exclusion criteria or because the patients no longer wished to participate (see patient flowchart in Figure 1), and therefore 208 patients were included. Of the excluded patients, 380 had a length of stay of longer than 48 hours. There was no difference in underlying medical disease between the 208 included patients and the 599 excluded ones. The age of the 208 included patients did not differ from that of the 380 excluded ones who stayed for longer than 48 hours (mean [± standard deviation] age: 57 ± 16 years versus 54 ± 19 years), but their length of stay was longer (mean 21 ± 19 days versus 13 ± 18 days; *P *< 0.000001) and their severity of illness or injury was greater (SAPS II score: 44 ± 17 versus 37 ± 18; *P *< 0.0001). However, the reasons for their admission were similar, except for digestive tract pathologies because of digestive haemorrhages (which were exclusion criteria) and cancers (7/208 versus 35/380; *P *= 0.01).

Among the 208 included patients included, 102 received the probiotic strain (probiotic group). Most of the patients were hospitalized after surgery (29.4%), trauma (24.2%), or because of respiratory distress (11.4%). Except for sex (male:female ratio: 3.2 versus 1.8; *P *= 0.05), there were no significant differences between placebo and probiotic groups with respect to patient characteristics (age, severity of illness, length of stay, or median durations of gastric tube, catheter use, tracheal intubation or mechanical ventilation [including both invasive and noninvasive methods]; Table 1). Omeprazole (20 mg/day) was administered as standard stress ulcer prophylaxis to 90 (84.9%) and 93 (91.2%) patients in the placebo and probiotic groups, respectively. There was no main difference between the antibiotics administered before and during the study (Table 1) except for fluconazole (administered to 26 patients in the placebo group and to 45 patients in the probiotic group; *P *= 0.006) and, before isolation of *P. aeruginosa*, imipenem (7 versus 16 patients; *P *= 0.04) and ciprofloxacin (28 versus 40; *P *= 0.05). *L. casei rhamnosus *was detected in gastric aspirate from 52 patients in the probiotic group. The median length of stay before detection was 13 days (95% CI 9 days to 17 days). In these patients, *L. casei rhamnosus *was detected for (mean ± standard deviation) 49.6% ± 24.9% of the length of stay. The median bacteria concentration was 10^3^/ml (range 10^2^/ml to 10^6^/ml). No patient contracted *Lactobacillus *associated infection during the study.

**Table 1:** Characteristics of patients enrolled in the study

Characteristic	Placebo group (n = 106)	Probiotic group (n = 102)
Age (years; median [range])	57 (18–80)	60 (18–91)
Sex (male/female)	81/25^a^	65/37^a^
SAPS II score (mean ± SD)	44.2 ± 15.3	44.6 ± 16.0
Stay (days; median [range])	13.5 (3–88)	14.0 (3–91)
Gastric tube (days; median [range])	11.0 (2–88)	12.0 (1–90)
Urinary tract catheter (days; median [range])	13.0 (3–88)	14.0 (2–90)
Tracheal intubation (n^b^/days; median [range])	100/9.0 (1–88)	96/12.0 (1–90)
Ventilatory assistance (n^b^/days; median [range])	103/13.0 (3–88)	99/14.0 (2–90)
Vascular catheter (n^b^/days; median [range])	105/14.0 (3–88)	100/14.5 (2–90)
Antibiotic treatment	105	101
Antipseudomonas drugs (n; ceftazidime and/or imipenem and/or ciprofloxacin)	43	55
Fluconazole (n)	26^a^	45^a^

^a^*P *< 0.05. ^b^The total number is indicated for characteristic when different from 106 and 102 for the placebo and the probiotic group, respectively. SAPS, Simplified Acute Physiology Score; SD, standard deviation.

Six and three patients of the placebo and probiotic group, respectively, acquired gastric *P. aeruginosa *(Table 2). Only three (2.8%) and one (1.0%) of these patients in the placebo and probiotic groups, respectively, had acquired ceftazidime-resistant isolates. Three patients in the probiotic group had concomitant Lcr35 and *P. aeruginosa *isolates in gastric aspirates. No statistically significant differences were observed between the two groups in the delay to gastric acquisition of *P. aeruginosa *(see Table 2).

**Table 2:** Incidence of *Pseudomonas aeruginosa *isolates among patients

	Gastric aspirate		Respiratory	
	
	Placebo group	Probiotic group	Placebo group	Probiotic group
Number of patients acquiring *P. aeruginosa *during the stay	6	3	13	5
Median time before acquisition (days [range])	30 (6–53)	16 (6–18)	11^a ^(5–40)	50^a ^(11–75)
NAE mean (95% CI)	73 (60–85)	87 (82–92)	69^a ^(59–79)	77^a ^(67–87)
% NA at day 21	97.4	94.7	85.3	98.5
% NA at day 42	84.4	94.7	70.9	93.3

^a^*P *< 0.05. CI, confidence interval; NA, not acquired; NAE, non acquisition expectancy.

**Figure 2:**
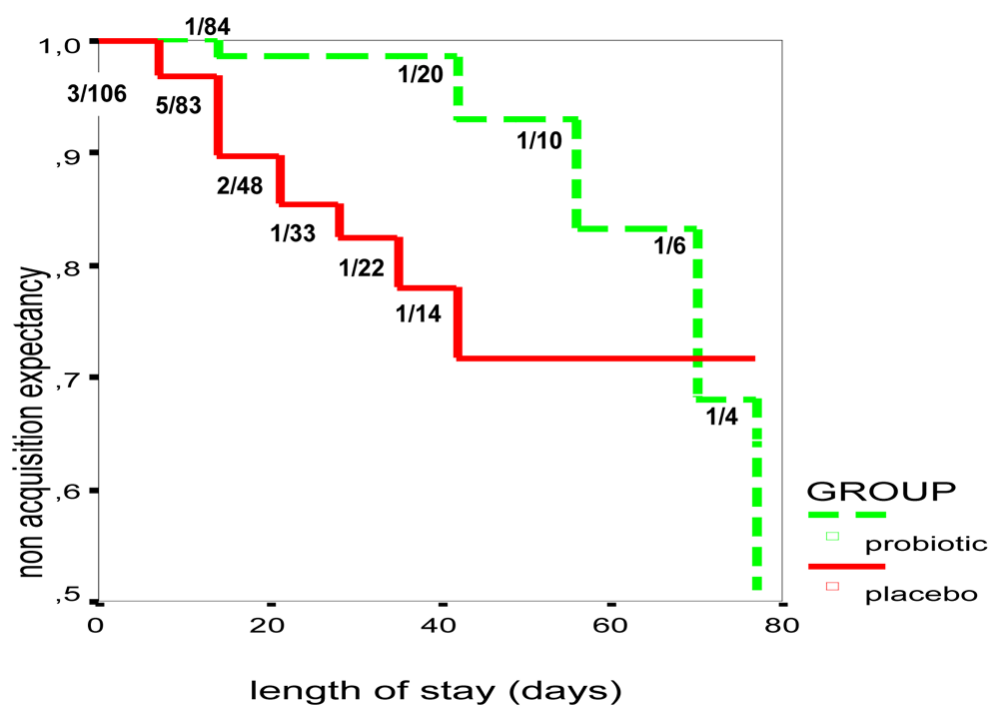
Actuarial representation of estimated probabilities of non-acquisition of *Pseudomonas aeruginosa *in the respiratory tract. The numbers of patients acquiring *P. aeruginosa *relative to the number of patients under study are indicated directly at each time point.

From the respiratory tract specimens, 13 positive samples – including five ceftazidime-resistant isolates – were detected in the placebo group and only five (all ceftazidime susceptible) in the probiotic group. A significant difference between groups was observed in acquisition delay for this pathogen (Figure 2), which was not related to any difference in prior treatment with this antibiotic (14 versus 16 patients in placebo and probiotic groups, respectively, received ceftazidime). *P. aeruginosa *was also responsible for VAP in eight patients in the placebo group and three in the probiotic group; this difference was not statistically significant. The median delays to *P. aeruginosa *VAP acquisition were shorter for the placebo group than for the probiotic group.

Respiratory tract colonization/infection did not strictly occured in patients positive for gastric colonization. *P. aeruginosa *was isolated from both gastric and respiratory specimens from only two patients in the placebo group and two in the probiotic group. Regarding the time of acquisition in these patients, the gastric specimens were positive before the respiratory tract samples were for all of them but one. When considering patients having a *P. aeruginosa *isolate in one or both of the two specimen types, either gastric or respiratory, 17 patients in the placebo group and six patients in the probiotic group were positive (*P *= 0.02).

During the course of the study, patients enrolled in the two groups also acquired nonpseudomonal infections. Indeed, VAP due to Enterobacteriaceae or *Staphylococcus aureus *were observed, but the differences between the two groups were not statistically significant (five cases in the placebo group versus nine in the probiotic group, and 11 in the placebo group versus 12 in the probiotic group for infections due to Enterobacteriaceae and *S. aureus*, respectively). In addition, isolation of *Candida *spp. from the respiratory tract did not differ significantly (seven patients versus 13 patients). Urinary tract, catheter-related and bloodstream infections were also observed, but their frequencies were not statistically different between the two groups (data not shown).

By univariate Cox regression analysis, we found that the variables that differed between the two groups were not associated with *P. aeruginosa *respiratory infection and/or colonization. However, three confounding variables – absence of probiotic treatment, weight and amoxicillin-clavulanate treatment (*P *< 0.10) – were identified (Tables 3 and 4). Because of the low number of patients with *P. aeruginosa *infection/colonization, they were included by pair in the multivariable Cox regression model [37]. In these analyses, weight (adjusted hazard ratio = 1.02, 95% CI = 1.00 to 1.1) and the absence of probiotic treatment (adjusted hazard ratio = 3.2, 95% CI = 1.1 to 9.1) were found to be independent factors associated with increased risk for *P. aeruginosa *respiratory infection and or colonization (Table 5).

**Table 3:** Simple Cox analysis indicating risk factors for *P. aeruginosa *respiratory infection and/or colonization

Variable	n	*P. aeruginosa *(n [%])	Hazard ratio	95% CI	*P*
Female sex	62	4 (6.5%)	0.71	0.23–2.15	0.54
Absence of probiotic	106	13 (12.3%)	3.31	1.17–9.40	0.024
Hospitalization duration < 15 days	107	4 (3.7%)	0.59	0.15–2.34	0.45
SAPS II score < 43	105	6 (5.7%)	0.69	0.26–1.85	0.46
Traumatology and surgical pathology	126	11 (8.7%)	0.97	0.38–2.51	0.95
Underlying respiratory tract disease	33	5 (15.2%)	1.63	0.58–4.58	0.35
Amoxicillin/clavulanate	120	13 (10.8%)	0.41	0.14–1.20	0.10
Piperacillin + tazobactam	21	3 (14.3%)	1.24	0.36–4.33	0.73
Cefotaxime	54	7 (13.0%)	1.15	0.44–3.01	0.77
Cefepime	42	6 (14.3%)	1.03	0.38–2.80	0.96
Ceftazidime	33	7 (21.2%)	1.59	0.59–4.27	0.35
Imipenem	30	6 (20.0%)	1.05	0.36–3.03	0.93
Aminoglycosides	20	1 (5.0%)	0.28	0.04–2.14	0.22
Ciprofloxacin	74	8 (10.8%)	0.59	0.22–1.59	0.30
Quinolones	138	14 (10.1%)	0.66	0.21–2.09	0.48
Glycopeptides	59	10 (16.9%)	1.42	0.54–3.76	0.47
Fluconazole	71	8 (11.3%)	0.56	0.21–1.50	0.25
Enterobacteriaceae infection/colonization	91	9 (9.9%)	0.88	0.35–2.24	0.79
*Candida *respiratory tract colonization	20	2 (10.0%)	0.60	0.13–2.65	0.50
*S. aureus *respiratory tract infection/colonization	39	8 (20.5%)	1.69	0.65–4.39	0.28

CI, confidence interval; SAPS, Simplified Acute Physiology Score.

**Table 4:** Simple Cox analysis indicating risk factors for *Pseudomonas aeruginosa *respiratory and/or colonization

Variable	Patients without *P. aeruginosa *in respiratory tract (n = 190)	Patients with *P*. aeruginosa in respiratory tract (n = 18)	Hazard ratio	95% CI	*P*
Age (years; mean ± SD)	56.7 ± 16.3	62.8 ± 15.0	1.02	0.97–1.05	0.48
Weight (kg; mean ± SD)	75.3 ± 16.9	83.9 ± 16.6	1.03	1.00–1.06	0.023

CI, confidence interval; SD, standard deviation.

**Table 5:** Multivariate Cox regression model showing independent factors associated with *Pseudomonas aeruginosa *respiratory infection/colonization

Variable	Adjusted hazards ratio	95% CI	*P*
No receipt of probiotic	3.17	1.11–9.08	0.032
Previous treatment with amoxicillin + clavulanate	0.43	0.15–0.14	0.18

CI, confidence interval.

## Discussion

The goal of the present study was to determine whether oral administration of a well known probiotic, namely Lcr35, could prevent colonization of the stomach with the pathogen *P. aeruginosa*, and therefore inhibit the development of an infectious process. We previously demonstrated that Lcr35 can adhere to intestinal cells and transiently colonize the intestinal tract of humans [24,25]. In addition, *in vitro *assays had demonstrated an inhibitory effect of Lcr35 on the growth of both Gram-positive cocci and Gram-negative bacilli, including *P. aeruginosa *[25]. Because our aim was to prevent pathogen overgrowth in the stomach, Lcr35 was administered as a powder through a nasogastric tube, and so this study differs from previous ones, which aimed to modify the intestinal flora [2]. Previous *in vivo *studies conducted in voluntary humans demonstrated that *L. rhamnosus *colonized the intestinal tract when 10^8 ^CFUs/day were administered [24]; therefore, a dosage of 10^9 ^CFUs/12 hours was chosen.

Because of difficulties in recruiting patients and despite an increase in duration of the survey, the planned number of patients was not obtained. The SAPS II values indicate that the included patients were more seriously ill than were the non-included ones, and were therefore more susceptible to infection during their stay. We observed a significant difference in delay to *P. aeruginosa *colonization/infection, with a threefold increased risk for respiratory *P. aeruginosa *colonization and/or infection in the patients without administered Lcr35. This effect could be due to the fact that more patients in the probiotic group were treated with antibiotics with activity against *P. aeruginosa*, but no statistically significant relation has been identified between *P. aeruginosa *infection and these antibiotics. Although the numbers of *P. aeruginosa *strains isolated in our study are too small to draw any definitive conclusions, our findings showed that *P. aeruginosa *acquisition occurred later in patients in the probiotic group than in the placebo group, especially for respiratory tract specimens, with a delay to acquisition (mean 50 days) longer than the mean duration of stay of all patients from this group (14 days). In 18 patients in whom *P. aeruginosa *isolated from the respiratory tract, the inclusion of two variables in a multivariable Cox regression model corresponded to nine events per variable, which is very close to the lower recommended threshold (≥ 10), and the risk for not detecting a confounding variable is therefore low [37]. The hazard ratio observed for weight was very close to 1.0 (1.02), showing that – despite a significant relation – the influence of weight on *P. aeruginosa *respiratory tract infection and/or colonization was weak.

Hence, oral administration of probiotics could be an alternative for preventing colonization by this pathogen, which occurs mostly in the long-term care critically ill patients and might be a means of warding off contamination until ventilatory assistance can be withdrawn. Administration of probiotics is not expected to eradicate the pathogens as antibiotics would do, but delaying the time to colonization while the patients are receiving ventilatory assistance – and therefore highly likely to become colonized – could be beneficial. Nevertheless, this work has several limitations, and should be considered a pilot study; further analyses conducted in multicentre clinical trials are necessary to test the hypothesis, because the potential application of probiotics has been poorly investigated [38]. Applications of probiotics have mostly been limited to the treatment of intestinal disorders such as diarrhoea and inflammatory diseases [39-43]. There is mounting evidence that probiotics might also offer an alternative strategy to antibiotic gastric decontamination in the future. A decrease in the rate of postoperative infections was observed in patients receiving oral *L. plantarum *together with enteral fibre nutrition [41]. Similar effects were obtained in studies involving patients undergoing major abdominal surgery or suffering from acute pancreatitis [39,40]. In contrast, Anderson and coworkers [28] did not observe any measurable effect on gut barrier function when they administered a mixture of probiotic strains and prebiotics (nondigestible sugars that selectively stimulate the growth of certain colonic bacteria) in a randomized controlled trial conducted in surgical patients, whereas Spindler-Vesel and colleagues [44] reported fewer infections in critically ill trauma patients receiving synbiotics in a randomized study involving 113 patients.

Although probiotics have been widely used in food processing for many years and overall have an excellent safety record [45], one important area of concern with their use is the risk for sepsis. Several reports have directly linked cases of *Lactobacillus *sepsis in adults to the ingestion of probiotic supplements, but the sources of infection were not conclusively proven [46]. In our study, which did not include immunocompromised or debilitated patients, no case of *Lactobacillus*-related sepsis was observed. Lcr35 did not colonize the stomach of all patients in the probiotic group, because it was detected in the stomach of only 51% of those tested. Individual unknown factors such as composition of the endogeneous flora may explain why some patients were not colonized, because no statistical link was observed between Lcr35 colonization and antibiotic treatment.

It remains to be determined whether the effect observed in our study is species specific or would affect other pathogens whose multiplication can occur in the stomach. Regarding the rate of infections due to Enterobacteriaceae in the enrolled patients during the time of the present study, no major difference was observed (data not shown). This would indicate that the interactions between probiotic and pathogen are strain related; therefore, extended studies including other probiotics are required.

## Conclusion

Our findings suggest that the oral administration of a probiotic to prevent infectious complications must be evaluated. Generalization of these study findings may not yet be justified, because this study was conducted using only one probiotic strain and in a single medical-surgical ICU including a mixed population of medical, surgical, and trauma patients. However, the place of probiotics in the prevention of infectious complications in surgical or critically ill patients warrants further investigation.

## Key messages

• Preventative carriage of potentially pathogenic micro-organisms from the aerodigestive tract is an infection control strategy to reduce the occurrence of hospital-acquired infections.

• The barrier provided by probiotics (nonpathogens) could restrain pathogens by impairing the colonization of mucosal surfaces.

• Our observational study suggests that oral administration of the probiotic Lcr35 delayed respiratory tract colonization/infection by *P. aeruginosa*, but had no beneficial effect on the occurrence of *P. aeruginosa *VAP.

## Abbreviations

CFU = colony-forming unit; ICU = intensive care unit; Lcr35 = *Lactobacillus casei rhamnosus *strain 35; SAPS = Simplified Acute Physiology Score; SDD = selective decontamination of the digestive tract; VAP = ventilator-assisted pneumonia. CI: confidence interval

## Competing interests

The authors declare that they have no competing interests.

## Authors' contributions

CF, DG and CD participated in designing the study. CF, DG, VC and JS participated in collecting and entering data. CD performed the statistical analysis. All authors were responsible for critical analysis and interpretation of the data. All authors read and approved the final manuscript.
